# Rapid stimulation of cellular Pi uptake by the inositol pyrophosphate InsP_8_ induced by its photothermal release from lipid nanocarriers using a near infra-red light-emitting diode†
†Electronic supplementary information (ESI) available. See DOI: 10.1039/d0sc02144j


**DOI:** 10.1039/d0sc02144j

**Published:** 2020-09-08

**Authors:** Zhenzhen Wang, Nikolaus Jork, Tamara Bittner, Huanchen Wang, Henning J. Jessen, Stephen B. Shears

**Affiliations:** a Signal Transduction Laboratory , National Institute of Environmental Health Sciences , National Institutes of Health , Research Triangle Park , NC 27709 , USA . Email: shears@niehs.nih.gov ; Tel: +1-984-287-3483; b Institute of Organic Chemistry , CIBSS , Center for Integrative Biological Signalling Studies , University of Freiburg , 79104 Freiburg , Germany

## Abstract

Inositol pyrophosphates (PP-InsPs), including diphospho-*myo*-inositol pentakisphosphate (5-InsP_7_) and bis-diphospho-*myo*-inositol tetrakisphosphate (1,5-InsP_8_), are highly polar, membrane-impermeant signaling molecules that control many homeostatic responses to metabolic and bioenergetic imbalance. To delineate their molecular activities, there is an increasing need for a toolbox of methodologies for real-time modulation of PP-InsP levels inside large populations of cultured cells. Here, we describe procedures to package PP-InsPs into thermosensitive phospholipid nanocapsules that are impregnated with a near infra-red photothermal dye; these liposomes are readily accumulated into cultured cells. The PP-InsPs remain trapped inside the liposomes until the cultures are illuminated with a near infra-red light-emitting diode (LED) which permeabilizes the liposomes to promote PP-InsP release. Additionally, so as to optimize these procedures, a novel stably fluorescent 5-InsP_7_ analogue (*i.e.*, 5-FAM-InsP_7_) was synthesized with the assistance of click-chemistry; the delivery and deposition of the analogue inside cells was monitored by flow cytometry and by confocal microscopy. We describe quantitatively-controlled PP-InsP release inside cells within 5 min of LED irradiation, without measurable effect upon cell integrity, using a collimated 22 mm beam that can irradiate up to 10^6^ cultured cells. Finally, to interrogate the biological value of these procedures, we delivered 1,5-InsP_8_ into HCT116 cells and showed it to dose-dependently stimulate the rate of [^33^P]-Pi uptake; these observations reveal a rheostatic range of concentrations over which 1,5-InsP_8_ is biologically functional in Pi homeostasis.

## Abstract 2

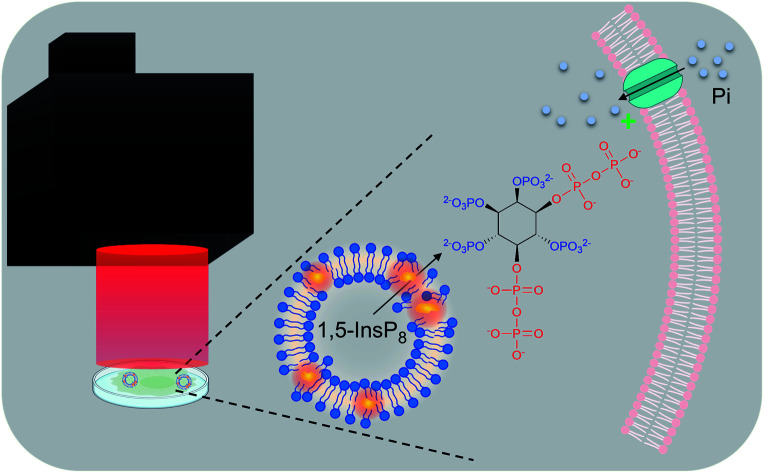
Thermosensitive liposomes were used to deliver inositol pyrophosphates (highly polar, cell-impermeant signaling molecules) into cultured cells; cargo release was induced within 5 min irradiation by a high power, near infra-red, light emitting diode.

## Introduction

**Fig. 1:**
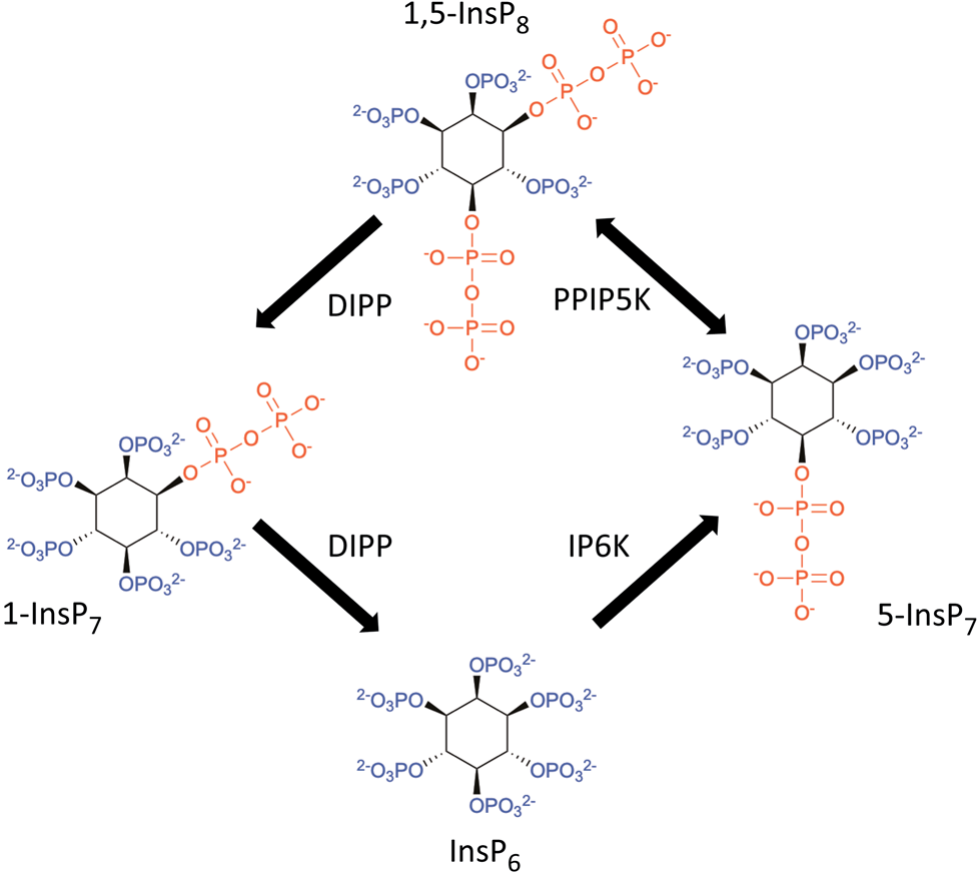
The primary pathways of 1,5-InsP_8_ synthesis and metabolism. The graphic incorporates a proposal^7^,^61^ that the pathway for 1,5-InsP_8_ turnover comprises a predominantly cyclical interconversion of InsP_6_, 5-InsP_7_, 1,5-InsP_8_, and 1-InsP_7_. IP6K = inositol hexakisphosphate 5-kinase; PPIP5K = diphosphoinositol pentakisphosphate 1-kinase (this enzyme also possesses an active 1,5-InsP_8_ 1-phosphatase activity); DIPP = diphosphoinositol polyphosphate phosphatase.

Diphosphoinositol polyphosphates (also known as ‘inositol pyrophosphates’, or PP-InsPs; Fig. 1) represent an extreme example of the application of phosphate esters and anhydrides as multipurpose cell-signaling entities: the PP-InsPs contain up to seven or eight phosphates that are tightly clustered around the six-carbon inositol ring. These polyphosphate assemblies represent a thermodynamically favorable springboard from which a β-phosphate can be non-enzymatically transferred to a preexisting phospho-serine residue that lies within an appropriately acidic protein microenvironment;^1^,^2^ this unique mechanism of covalent modification – ‘pyrophosphorylation’^2^ – regulates the functions of a number of proteins that control diverse biological processes.^3^ In addition, the chemical synthesis and application of non-hydrolysable methylene bisphosphonate (PCP-) bioisosteres of the PP-InsPs has helped to demonstrate that this class of cellular signals also regulate cellular physiology through alternate, allosteric modification of protein activities.^4^–^6^ The biological consequences of both of these mechanisms of action of PP-InsPs are being studied in yeasts, plant and animal cells, and there is a growing focus on their regulation of metabolic balance, particularly with regards to cellular homeostasis of inorganic phosphate (Pi).^5^,^7^–^12^

PP-InsP biology in cultured cells and animal models has typically been studied by genetic manipulation of the levels of enzymes that synthesize and catabolize these polyphosphates. For example, knockout of the *vip1* gene in *Saccharomyces cerevisiae* in order to prevent 1-InsP_7_ synthesis has been taken as genetic evidence that this PP-InsP normally attenuates Pho81 activity as an inhibitor of Pho80–Pho85 cyclin/cyclin-dependent kinase activity.^13^ However, there is an alternative genetic rationale for the latter result, that need not involve 1-InsP_7_ synthesis: Pho81 expression has been reported to be non-specifically reduced in *vip1*Δ cells.^14^ This situation underscores how unsuspected off-target changes in gene expression can undermine the conclusions derived solely from genetic experiments.

One other experimental option for studying actions of PP-InsPs is to block their synthesis with inhibitors of inositol phosphate kinases, but currently there is only one such research tool in common use: the pan-IP6K inhibitor, *N*2-(*m*-(trifluoromethyl)benzyl) *N*6-(*p*-nitrobenzyl)purine (TNP).^15^ While a number of published reports attribute the biological effects of TNP to reduced synthesis of the IP6K product, 5-InsP_7_, this reagent also inevitably inhibits 1,5-InsP_8_ synthesis (Fig. 1, and see ref. 5), so the drug cannot by itself be used to distinguish the biological actions of 5-InsP_7_ from those of 1,5-InsP_8_. Furthermore, TNP competes with the binding of ATP to IP6K, and structural similarities between the nucleotide-binding sites of IP6Ks and protein kinases invite off-target effects of this inhibitor.^16^,^17^

Since the toolkit for probing PP-InsP actions is so limited, there is a particular need to study their functions by delivering these intracellular signals into intact cells. Unfortunately, the phosphorylated nature of the PP-InsPs prohibits them from crossing the plasma membrane. Microinjection offers one solution, but that is technically challenging and also not compatible with work on populations of cultured cells. Cell-permeant and bioactivatable analogs of inositol phosphates have been used successfully,^18^,^19^ but such molecules lack spatial selectivity, because they will penetrate into subcellular organelles. Temporal control is also limited, although that particular issue can be circumvented by attaching ‘caging’ groups that can be near-instantly unlocked by a laser, typically using UV-wavelengths.^20^ Laser-induced uncaging has powerful spatial and temporal resolution, but it also has two key limitations: safety concerns, and the limited spot-size, which is not appropriate for biological assays that require large numbers of cultured cells. The latter restriction can be circumvented by using an UV-emitting arc lamp^21^,^22^ but there are still safety concerns. Also, arc lamps have a limited lifetime, their power-output deteriorates during use, and they do not deliver a collimated beam with uniform intensity;^23^ such issues impede establishment of consistency of application between different laboratories. Light emitting diodes (LEDs) are not encumbered with these disadvantages, but nevertheless, UV-uncaging generates radicals, which are potentially damaging to the cells.^24^,^25^

An alternate approach has been to employ biocompatible nanoparticles as delivery vehicles. For example, guanidinium-rich oligocarbonate transporters^26^ have proven to be successful. Liposomes have also been used to deliver a number of polyphosphorylated molecules into cells; appropriate lipid compositions have been described that facilitate endocytic liposomal entry into the cell, and then during the following 2–4 hours, fusion of the liposomal and endosomal membranes permits slow and gradual escape of the encapsulated cargo into the cytosol. This has enabled successful delivery into cells of microRNAs,^27^ siRNA,^28^ antisense DNA,^29^ and ATP.^30^,^31^ We recently used this technique to transfer PP-InsPs into cells.^5^ However, the hours-long timeframe required for spontaneous cargo release by nanoparticles is not compatible with the study of fast-acting signal transduction processes. Moreover, the metabolism of the released PP-InsP becomes a complicating issue during this lengthy procedure. While this specific issue can be circumvented by delivering metabolically stable PP-InsP analogues into cells,^6^,^32^–^34^ such molecules cannot support protein pyrophosphorylation, and so these bioisosteres do not recapitulate all PP-InsP functions. Thus, for this study, we turned our attention to a more rapid cargo-release mechanism – photolysis of dye-sensitized, thermolabile liposomes^24^,^25^,^35^,^36^ – that is more compatible with the use of natural PP-InsPs.

These photothermal procedures utilize liposomes with a composition that facilitates a gel to liquid transition at temperatures just above 37 °C. For example, dipalmitoylphosphatidylcholine (DPPC) exhibits a transition temperature (*T*_m_) of 41 °C. As DPPC liposomes are heated, increased leakiness due to lipid ‘melting’ can occur somewhat below the *T*_m_, through the formation of permeable, disordered regions at gel/liquid boundaries.^37^ By doping the DPPC with another phospholipid with a lower *T*_m_ (such as 1,2-dioleoyl-*sn*-glycero-3-phosphocholine (DOPC)), not only is the net *T*_m_ slightly lowered, there are also nanodomains in DPPC : DOPC mixtures that facilitate increased formation of permeable, interfacial boundaries.^37^,^38^ This temperature-induced increase in liposome permeability has previously been exploited for photothermal release of InsP_3_ delivered into CHO-M1 cells.^24^ In the latter study, liposomes were fabricated from a 9 : 1 v/v mixture of DPPC : DOPC doped with a near infrared and photothermal carbocyanine dye, which was laser-activated to ‘melt’ the vesicles and release the cargo.^24^

Due to the inherent limitations of laser usage (see above), we have instead optimized the thermolytic approach using an LED that emits a collimated and near-infra red beam with a 22 mm diameter. Such long wavelength light easily penetrates biological materials and does not generate radicals, unlike the UV light that is frequently used for uncaging.^25^ Nevertheless, we considered that the intense polarity of PP-InsPs could have made their encapsulation and delivery particularly challenging. The PP-InsPs also exhibit anomalously restricted diffusion properties,^39^ which we were concerned might hinder their escape from leaky vesicles. Thus, we had a particular need to quantify the efficiency of our technique; this we accomplished by synthesizing a novel, stably fluorescent PP-InsP analogue.

Fluorescent probes have previously been attached to inositol phosphates, for example by conjugating fluorescein derivatives to the 2-OH group of Ins(1,4,5)P_3_ (ref. 40) and InsP_5_ (ref. 41). However, there are different chemical challenges to the attachment of any probe to a phosphate group, which is required for synthesis of PP-InsP analogues.^42^ A recent report^21^ describes the synthesis of a 5-InsP_7_ analogue containing [7-(diethylamino)coumarin-4-yl]methyl (DEACM) groups, for the purpose of UV (375 nm) driven uncaging. Naturally, the weakly fluorescent DEACM moieties are released from the PP-InsP during the uncaging. Thus, no stably fluorescent version of a PP-InsP with a high quantum yield has previously been described. Here, we report the first clickable 5-InsP_7_ with an alkyne attached to the β-phosphate, and we demonstrate that these highly polar species efficiently undergo Huisgen 1,3-dipolar cycloaddition with azides in the presence of copper(i).^43^ This is the first example of the application to PP-InsP research of the ample opportunities of click chemistry, opening up access to additional, diverse probes. The value of 5-FAM-InsP_7_ as a new research tool is highlighted by our using it to establish a new intracellular PP-InsP delivery method that is quantitatively tunable, and also applicable to all available PP-InsPs and analogs.

## Results and discussion

### Synthesis of 5-FAM-InsP_7_

**Scheme 1:**
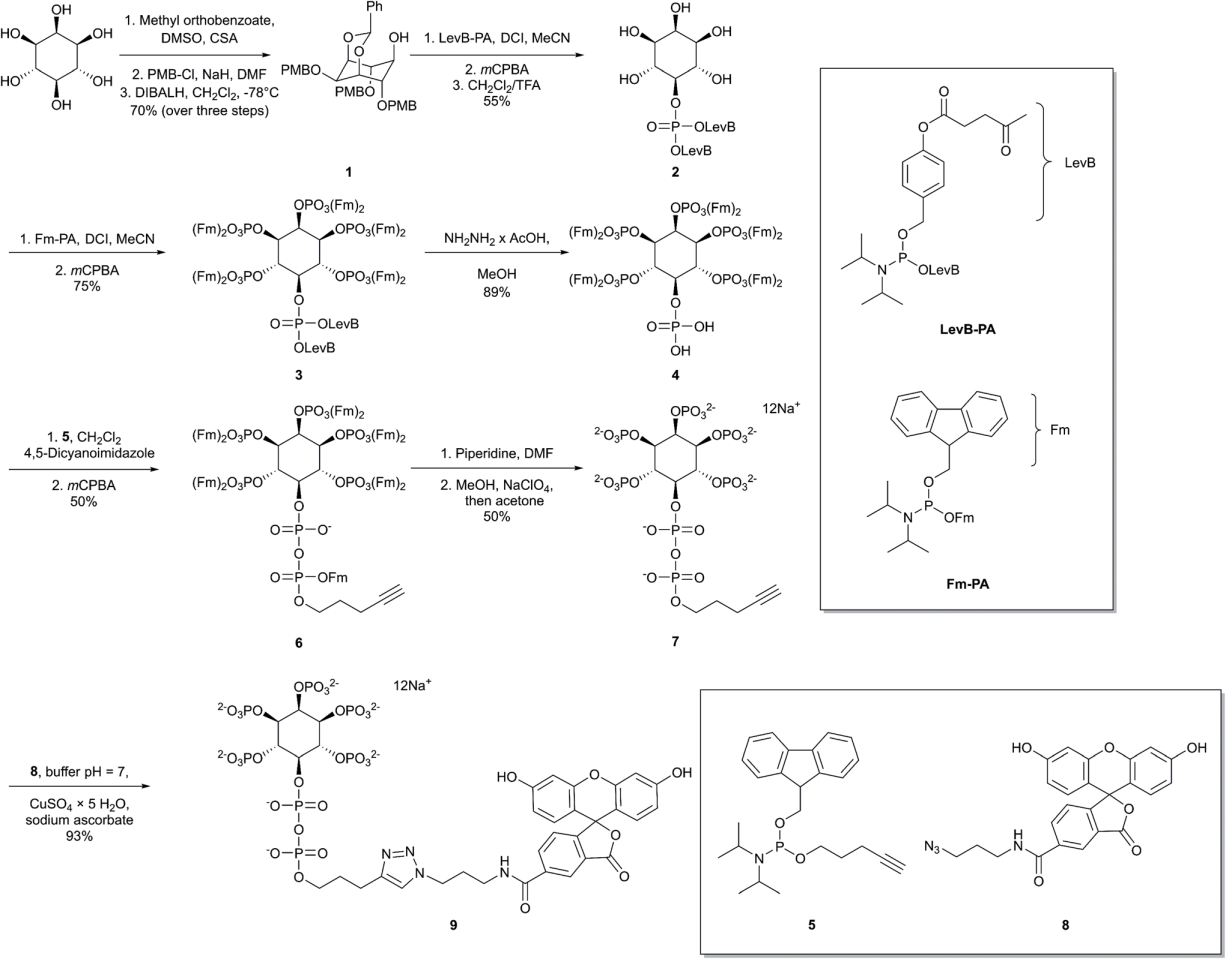
Synthesis of fluorescein labelled 5-InsP_7_**9** (5-FAM-InsP_7_). *myo*-Inositol was transformed into the target molecule **9** in a sequence involving P-anhydride formation, removal of protecting groups, and azide–alkyne dipolar cycloaddition with FAM-azide **8**.

**Fig. 2:**
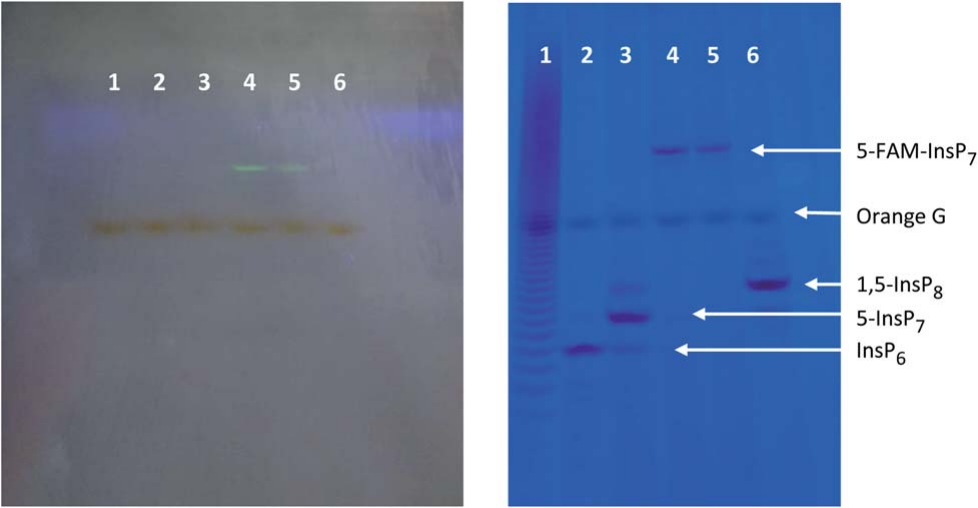
PAGE of 5-FAM-InsP_7_ and inositol polyphosphate standards. The left-hand image was obtained after illumination at 365 nm, prior to staining with toluidine blue. The gel on the right was stained with toluidine blue. Lane 1: PolyP_22_ (2 nmol). Lane 2: InsP_6_ (2.5 nmol). Lane 3: 5-InsP_7_ (1.25 nmol). Lanes 4 & 5: different batches of 5-FAM-InsP_7_ (3 nmol). Lane 6: 1,5-InsP_8_ (3 nmol).

Our first objective was to chemically synthesize a stably fluorescent PP-InsP analogue in order to quantify the delivery of PP-InsPs into intact cells, and hence maximize its efficiency. The synthesis (Scheme 1) commenced with commercially available *myo*-inositol. In order to install an alkyne on the diphosphate unit, we took recourse to our previously developed protecting group strategy:^44^ PMB and benzylidene acetal protected *myo*-Ins with a free OH group in the 5 position **1** is available in only three steps. Introduction of a LevB protected phosphate, followed by cleavage of PMB groups and the acetal in a single operation leads to selective installation of an orthogonally protected phosphate triester in the 5 position in compound **2**. Exhaustive phosphitylation with a fluorenylmethyl (Fm) modified phosphoramidite^45^ and oxidation yields protected hexakisphosphate **3**. The LevB groups can now be removed selectively using hydrazine acetate, releasing the free phosphate in the 5-position. Next, Fm protected and alkynylated P-amidite **5** was used to generate the phosphoric anhydride, proceeding *via* a mixed P(iii)–(v) intermediate followed by oxidation to the P(v)–(v) anhydride with *m*CPBA.^46^,^47^ Labeled anhydride **6** was obtained as a mixture of diastereomers, which converged to single alkynylated 5-InsP_7_**7** upon deprotection of the 11 Fm groups under basic conditions. The initially obtained piperidinium salt was transformed into the sodium salt by precipitation with sodium perchlorate in acetone.^48^ The water-soluble sodium salt was subjected to a 1,3 dipolar cycloaddition using copper catalysis in buffered water in over 90% isolated yield, demonstrating that copper catalysis is compatible with these densely phosphorylated structures. The fluorescent target molecule 5-FAM-InsP_7_**9** was analyzed by polyacrylamide gel electrophoresis (PAGE;^49^Fig. 2), revealing a single fluorescent band migrating more slowly than the reference compounds (InsP_6_, 5-InsP_7_, 1,5-InsP_8_) and orange G dye.

### Preparation and characterization of a photothermal liposomal delivery system *in vitro*

We used a lipid-film hydration/extrusion method (see Materials and methods) to construct thermolabile liposomes comprising a 9 : 1 : 1 ratio (by weight) of dipalmitoylphosphatidylcholine (DPPC): 1,2-dioleoyl-*sn*-glycero-3-phosphocholine (DOPC): 1,1′-dioctadecyl-3,3,3′,3′-tetramethylindotricarbocyanine iodide (DiR/NIR-780). This composition is similar to that employed in an earlier study^24^ except that an alternative carbocyanine dye with a shorter adsorption wavelength was used. The DiR we have used has negligible chemical cytotoxicity and strong light absorption that peaks at 780 nm.^36^

**Fig. 3:**
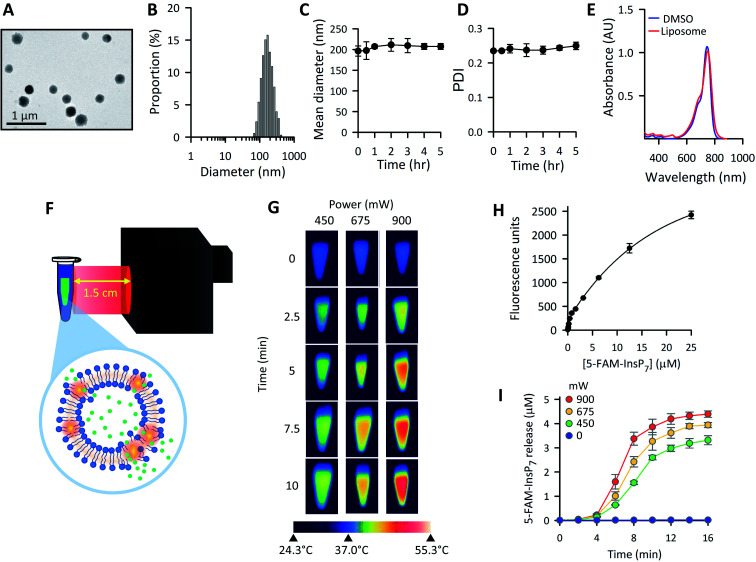
The preparation and characterization of thermolabile liposomes *in vitro*. (A) Representative TEM image of the liposomes. (B) Particle size distribution histograms of freshly prepared liposomes in culture medium (see Materials and methods). (C) Mean diameter and (D), polydispersity index (PDI), for liposomes that were incubated in culture medium at 37 °C for the indicated times. (E) UV-vis absorption spectra of either 6.25 μg mL^–1^ DiR in 1 mL DMSO (blue) or 6.25 μg mL^–1^ DiR incorporated into 50 μg mL^–1^ liposomes suspended in 1 mL PBS (red). (F) Graphic depicting the LED-mediated photothermal activation of liposomes *in vitro*; the green symbols represent the PP-InsP cargo that is released as the liposomes undergo a temperature-induced phase transition. (G) Thermal images of LED-illuminated liposomes. Centrifuge tubes containing 200 μg mL^–1^ liposomes, suspended in 1.0 mL PBS, were pre-warmed to 37 °C for 10 min. Tubes were removed from the water bath and illuminated at ambient temperature (24.3 °C) by the LED at the indicated power outputs and time points. Each image was obtained from a separate tube, which was then discarded. (H) Concentration-dependent fluorescence of 5-FAM-InsP_7_ in 5 mM HEPES, pH 7.0. (I) Liposome suspensions (200 μg mL^–1^ in 1.5 mL of 5 mM HEPES, pH 7.0) were irradiated at ambient temperature with the indicated LED power outputs and time points. The release of 5-FAM-InsP_7_ was monitored from the photothermal-induced increase in fluorescence caused by its unquenching as the molecule diffuses out of the liposomes, using the calibration data in panel H. Theoretical maximum 5-FAM-InsP_7_ release is equivalent to a concentration of 7 ± 0.1 μM (*n* = 3). Data in panels A, B, E and G are from representative experiments; data presented in panels C, D, H and I are means ± SD from 3 replicates.

Variations in liposome size can influence the kinetics and efficiency of cargo delivery into cells,^50^ consequently it is important to establish vesicle morphology. The spherical nature of our liposomes was revealed by using transmission electron microscopy (Fig. 3A). The ζ-potential of the liposomes was determined to be 7.6 ± 0.7 mV. Through dynamic light scattering analysis (Fig. 3B–D) we determined the liposome hydrodynamic size to be 197 ± 12 nm (mean ± SD; *n* = 3), with a polydispersity index (PDI) value of 0.24 ± 0.01, which is generally considered suitable for maintaining consistency of cargo delivery parameters.^50^ Both the hydrodynamic size and the PDI were stable for at least 5 h when liposomes were suspended at 37 °C in culture medium (Fig. 3C and D). Absorbance data (Fig. 3E) demonstrate that DiR was near-quantitatively incorporated into the liposomes.

The graphic in Fig. 3F depicts our strategy to increase permeability of DiR-impregnated liposomes upon irradiation with near infra-red light (peak wavelength = 734 nm). To visualize the photothermal process, we prewarmed to 37 °C a number of tubes containing 1.0 mL of an aqueous dispersion of liposomes; tubes were then removed from the waterbath and illuminated at ambient temperature (approx. 24 °C) using the power outputs and time points indicated in Fig. 3G. Thermal imaging showed significant elevations in temperatures in proportion to the power of the irradiation (Fig. 3G). To investigate if a photothermal response was accompanied by release of 5-FAM-InsP_7_ from the vesicles, we exploited the phenomenon of signal self-quenching that is associated with high intravesicular concentrations of the fluorescent probe:^51^,^52^ as the 5-FAM-InsP_7_ leaks out of the vesicles and becomes diluted, the fluorescence is unquenched. A suitable calibration curve was constructed that allowed the extravesicular 5-FAM-InsP_7_ concentration to be quantified (Fig. 3H). At ambient temperature, we found significant cargo release in a manner that was dependent upon the power output from the LED (Fig. 3I). There was no leakage of 5-FAM-InsP_7_ from the liposomes in the absence of LED illumination (Fig. 3I). We have also found there was negligible leakage from liposomes incubated in cell culture medium at 37 °C for 5 h (0.35 ± 0.2%, *n* = 3). With all of this information in hand, we went on to characterize and optimize various parameters for LED-activated cargo delivery and release in intact cells.

### Optimization of the delivery and intracellular release of PP-InsPs into cultured cells using thermolabile liposomes

**Fig. 4:**
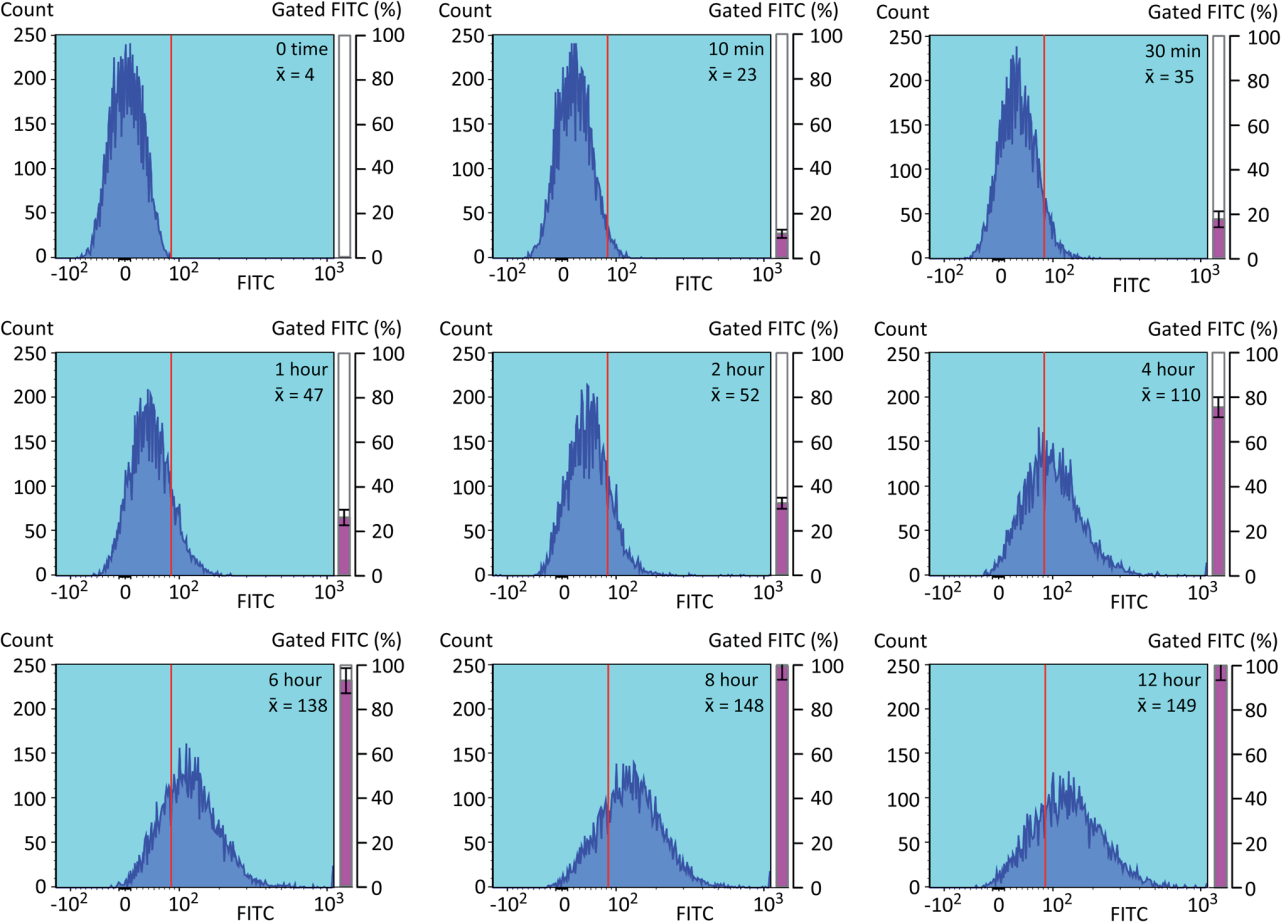
Analysis by flow cytometry of the influence of liposomal loading time upon LED-induced photothermal release of 5-FAM-InsP_7_ in intact cells. Liposomes (200 μg mL^–1^) were added to cultures of HCT116 cells (1.5 × 10^6^ per well) at 37 °C for the indicated loading times (see top right of each panel). Cells were then washed twice and fresh culture medium was added prior to LED illumination (450 mW; 3 cm distance; 10 min). Cells were then trypsinized, pelleted, and resuspended in 1 mL PBS, and the cellular fluorescence intensity was determined by flow cytometry (approx. 8000 cells per sample). A representative experiment is shown. The mean value for fluorescence intensity of the cell population is shown in the top right of each panel. The photothermally-induced increase in total cellular fluorescence above the value set by the gate (vertical red line) was normalized to 10 000 cells, and is presented as a percentage of the maximum values obtained (mean ± SEM) from 3 biological replicates; (vertical dark pink bars).

**Fig. 5:**
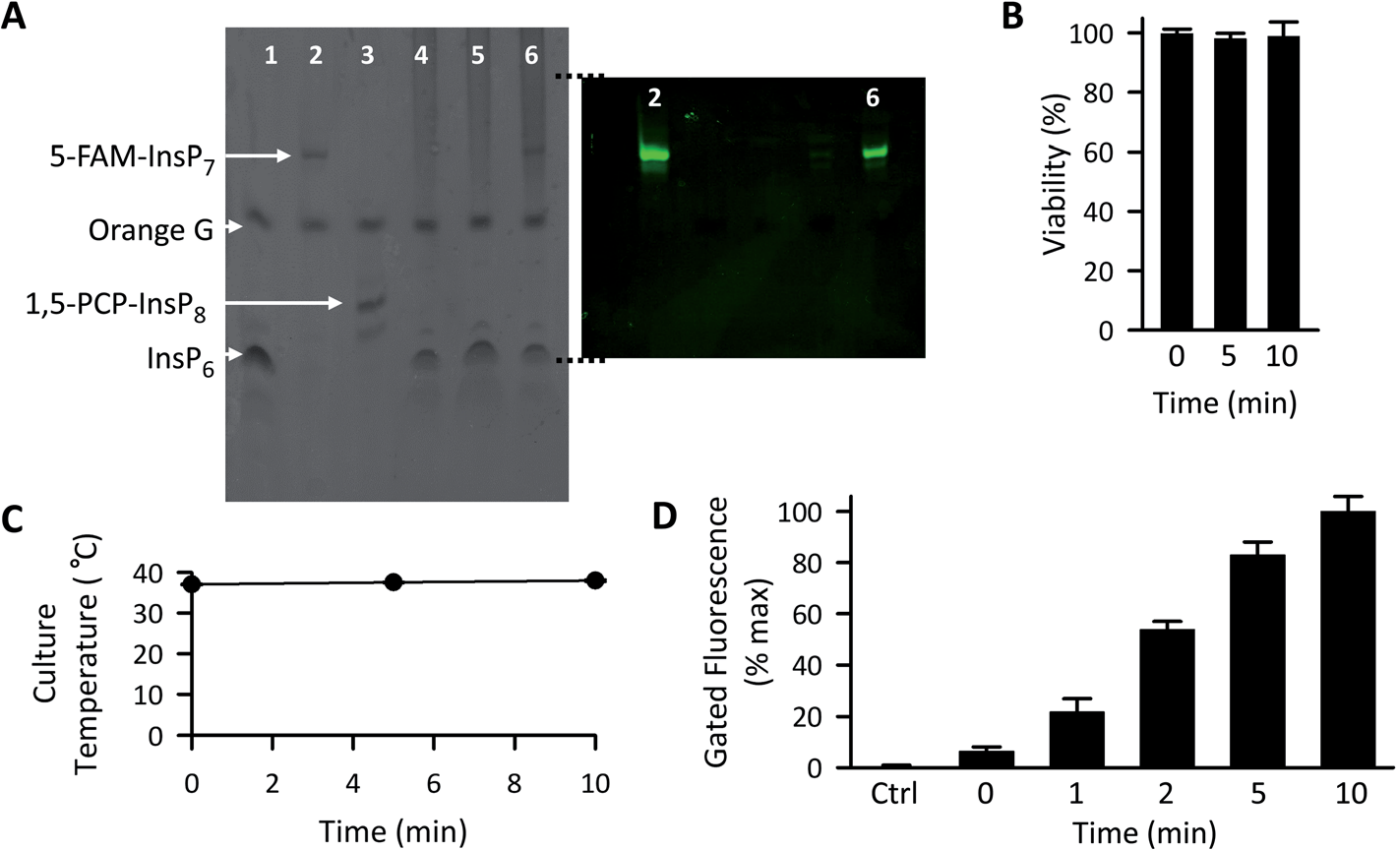
The effect of LED irradiation time upon intracellular 5-FAM-InsP_7_ release. (A) Representative PAGE analysis of 5-FAM-InsP_7_ incorporated into intact cells using liposomes. Two 150 mm plates, each containing approximately 5 × 10^7^ cells each cultured in 8 mL medium, were incubated with 120 μg mL^–1^ liposomes for 6 h. Cells were then acid-quenched, and polyphosphates were concentrated and analyzed by PAGE. Lane 1, 5 nmol InsP_6_ standard; lane 2, 5 nmol standard 5-FAM-InsP_7_; lane 3, 5 nmol standard 1,5-PCP-InsP_8_; lane 4, cell lysate; lane 5, lysate prepared from cells incubated with 2 nmol free 5-FAM-InsP_7_; lane 6, lysate prepared from cells incubated with liposomes containing 5-FAM-InsP_7_. The fluorescence in the gel was captured by using a GelDocXR+ (Bio-Rad). (B) Liposomes (200 μg mL^–1^) were added to cultures of HCT116 cells (1 × 10^4^ per well) for 4 h and then cells were washed twice and fresh culture medium was added prior to LED illumination (450 mW; 3 cm distance) for the indicated times. Then, 24 h later cell viability was tested using an MTT assay. (C and D) Cells were loaded with 200 μg mL^–1^ liposomes for 4 h as described for panel A, and then illuminated with the LED for the indicated times, and (see C) the temperature of the culture medium was monitored and (see D) after the indicated LED exposure times, cells were trypsinized, pelleted, and resuspended in 1 mL PBS, and the fluorescence intensity was determined by flow cytometry; data shown are for gated cells, determined as described in the legend to Fig. 4. Means and SEMs are provided from 3 biological replicates.

We next optimized the time of liposomal loading of PP-InsP cargo into cultured cells. Liposomes that contained 5-FAM-InsP_7_ were incubated with HCT116 cells for various loading times prior to 10 min of LED irradiation at a power output of 450 mW and a distance of 3 cm. Flow cytometry was used to record the intracellular fluorescence unquenching as 5-FAM-InsP_7_ was released from intracellular vesicles (Fig. 4). We determined the degree of background fluorescence in an equivalent number of control cells not loaded with liposomes; the cells in liposome-loaded cells were then gated based on a fluorescence value that excluded >99% of the control cells (vertical red lines in all panels of Fig. 4). Using this method, we established that approximately 80% of the maximum intracellular gain-of-fluorescence from LED irradiation was attained from 4 h of liposomal loading (vertical dark pink bars in Fig. 4, derived from 3 biological replicates). In a second approach, we simply measured the change in the mean of total fluorescence values from every cell, without correcting for background. Using this parameter, 4 h of liposomal loading was sufficient to attain approximately 75% of the maximum irradiation-dependent increase in the mean of total cellular fluorescence (see mean values in all panels in Fig. 4). Based on the high level of agreement between our two parameters, we decided to use 4 h of liposomal loading in the experiments described below. The liposomes are stable in culture medium during this time frame (Fig. 3C and D) and there is no significant 5-FAM-InsP_7_ leakage (see above). The 5-FAM-InsP_7_ that was extracted from the cells eluted as a single band upon PAGE analysis (Fig. 5A), indicating that there was no significant liberation of the FAM group during the loading procedure.

**Fig. 6:**
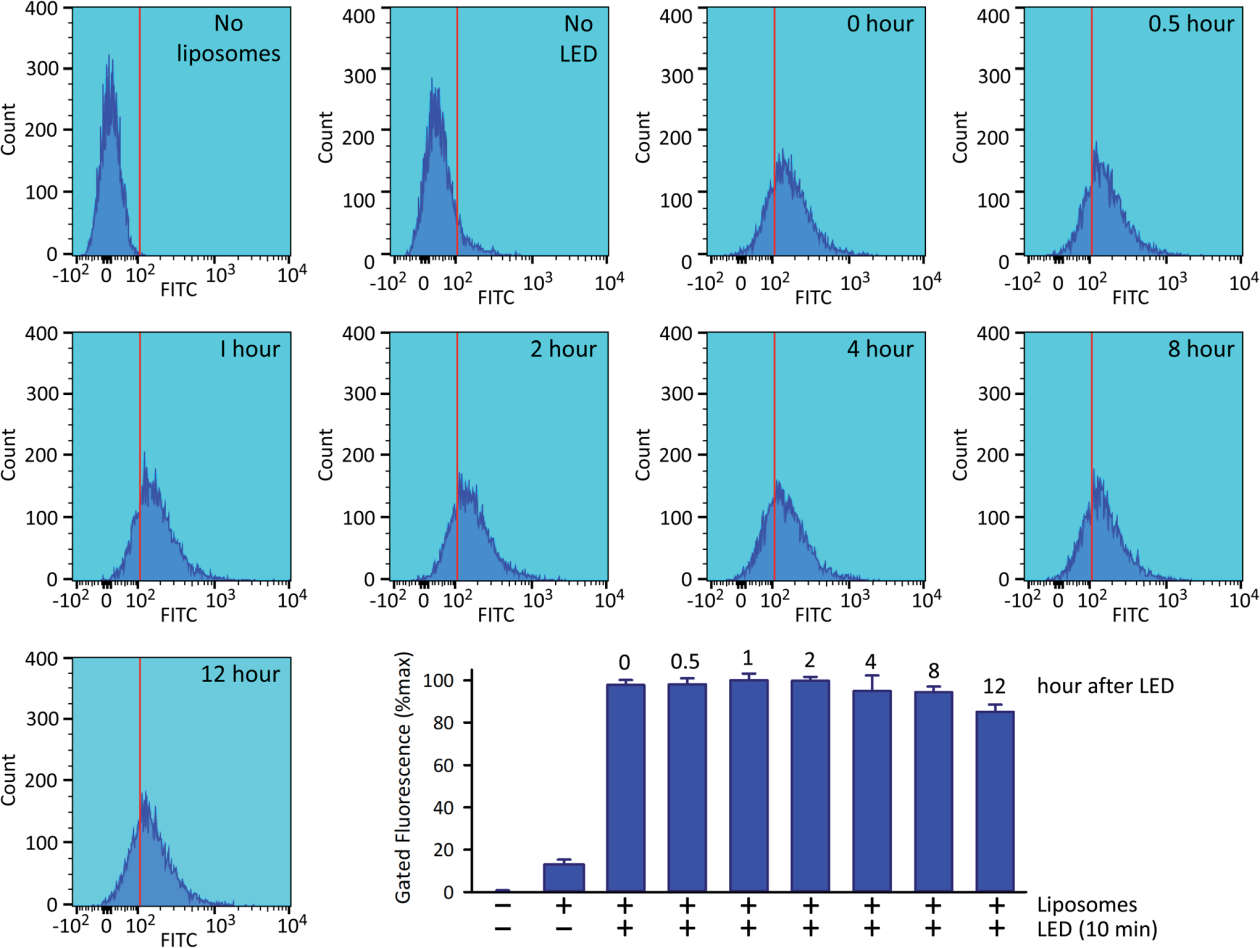
Stability of the intracellular 5-FAM-InsP_7_ signal. Exactly as described in the legend to Fig. 4, HCT116 cells were loaded with liposomes for 4 h and then washed twice and fresh culture medium was added prior to LED illumination for 10 min, and the cellular fluorescence intensity was determined by flow cytometry, either immediately (top row, second sample from the right, labeled as “0 hour”) or at the indicated subsequent times. The top row also describes two controls, one performed without liposomes, and one performed with liposomes but without LED irradiation. Each sample contained approx. 9000 cells. The bar graph summarizes the gated intracellular fluorescence from 3 biological replicates (means ± SEM).

Following 10 min exposure to the LED, there was no impact upon cellular integrity, as determined 24 h later by a colorimetric assay for reduction of 3-(4,5-dimethylthiazol-2-yl)-2,5-diphenyltetrazolium bromide tetrazolium (MTT; Fig. 5B). The temperature of the culture medium did not change during the illumination (Fig. 5C). When we investigated the relationship between the duration of the irradiation time and the degree of intracellular 5-FAM-InsP_7_ release, we found that considerable release was elicited after just 5 min illumination with the LED (80% of that at 10 min; Fig. 5D). Following release of the intracellular 5-FAM-InsP_7_ by 10 min LED irradiation, the fluorescence signal remained stable for at least 8 h (Fig. 6).

### Analysis by confocal microscopy of the intracellular disposition of 5-FAM-InsP_7_

**Fig. 7:**
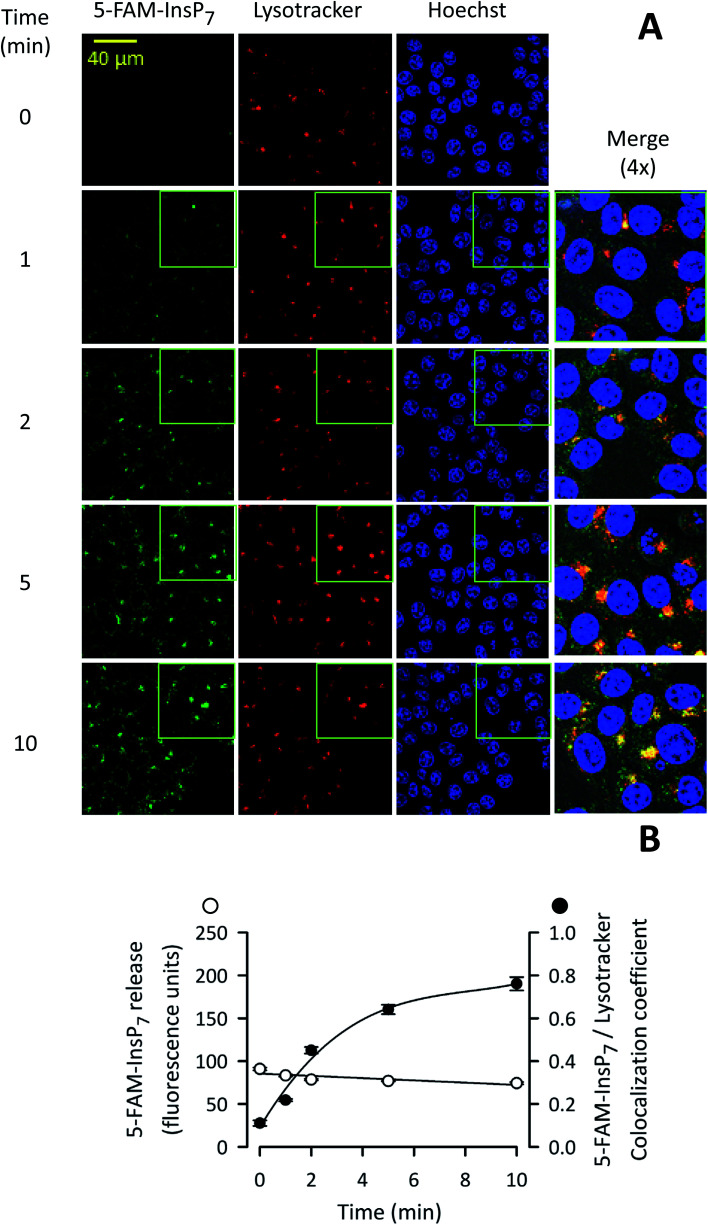
Analysis by confocal microscopy of the influence of time of LED irradiation of intact cells upon photothermal release of 5-FAM-InsP_7_. (A) Representative confocal images showing the impact of LED exposure time upon intracellular 5-FAM-InsP_7_ release. Liposomes (20 μg mL^–1^) containing 5-FAM-InsP_7_ were added to cultures of HCT116 cells (0.8 × 10^6^ per well; 1 mL medium; 4 h). Next, cells were washed twice in PBS and irradiated with LED (450 mW; 3 cm) for the indicated times. Cells were then stained with lysotracker and Hoechst. From left to right, the columns of panels show 5-FAM-InsP_7_ fluorescence (green), lysotracker staining (red), Hoechst staining (blue), and a 4× magnification signal merge; these data are representative images. (B) Mean values and standard deviations for total cellular 5-FAM-InsP_7_ fluorescence and the degree of lysotracker/5-FAM-InsP_7_ co-localization (determined at 63× the magnification of the wide-field images), derived from cells treated as described in panel A. Data were derived from 3 biological replicates (each of which involve analyzing 284–340 cells at every time point).

We next used confocal microscopy to study the intracellular distribution of photothermally-released 5-FAM-InsP_7_; the fluorescence signal was predominantly excluded from the nucleus (Fig. 7A). A minor proportion of released 5-FAM-InsP_7_ co-localized with LysoTracker red (Fig. 7A and B), a marker for lysosomes/endosomes. The weighted colocalization coefficient for the two fluorescent signals was 0.36 at zero time, decreasing to 0.30 during the experimental time course (Fig. 7B). Thus, we conclude there was a low degree of colocalization of the two signals. An analysis of the time course of the illumination-dependent increase in total intracellular fluorescence (Fig. 7B) showed that the value at 5 min was 80% of the value at 10 min: similar conclusions were drawn during analysis by flow cytometry (see above). These data indicate that our new methodology can be used to study acute signaling responses by PP-InsPs that require analysis of large populations of cultured cells.

### Photothermally-released PP-InsP is biologically active

**Fig. 8:**
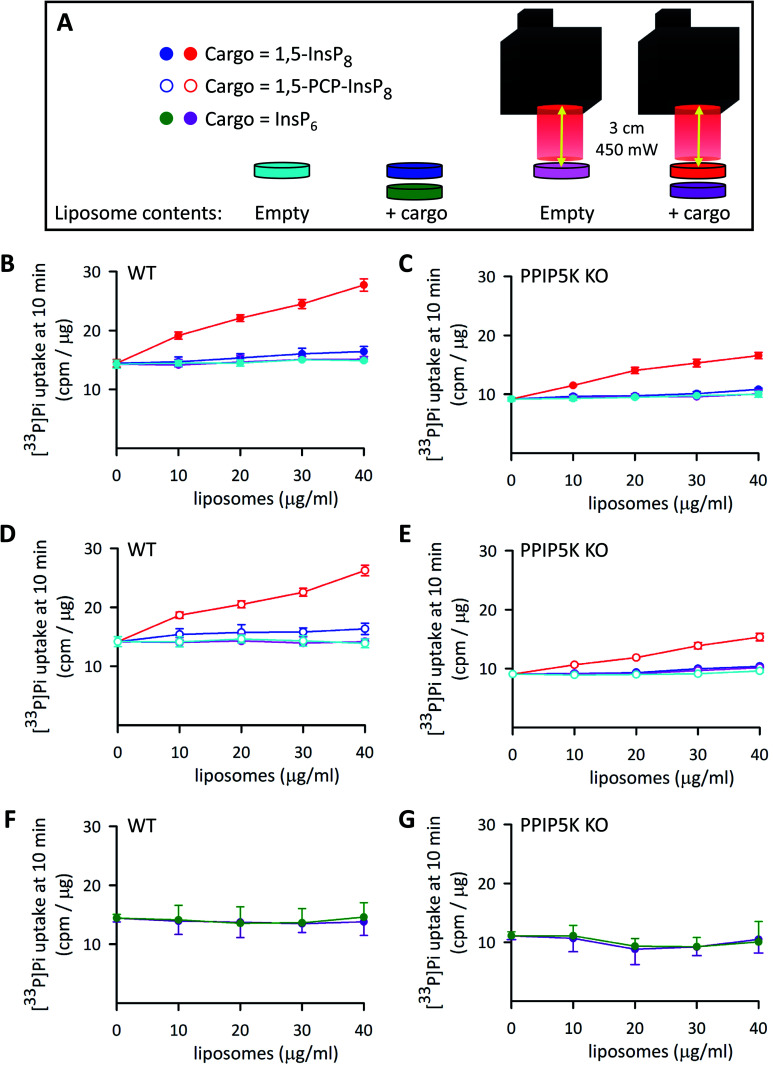
The effects of InsP_6_, 1,5-InsP_8_ and 1,5-PCP-InsP_8_ upon cellular Pi uptake. (A) Graphic depicting the experimental protocol. (B–G) Show [^33^P]-Pi influx from WT (B, D and F) and PPIP5K KO HCT116 cells (C, E and G). The cells (5 × 10^5^ per well in 12 well plates incubated at 37 °C) were treated for 4 h with either empty liposomes (cyan and pink symbols), or liposomes with cargo (filled red and dark blue symbols = 1,5-InsP_8_; open symbols, red and dark blue = 1,5-PCP-InsP_8_; dark green and purple symbols = InsP_6_). The liposome concentrations ranged from 10 to 40 μg mL^–1^ as indicated. Cells were then washed twice and fresh culture medium containing [^33^P]-Pi was added; the illumination protocol is shown in panel A. Data obtained from cultures that were irradiated with the LED (450 mW; 5 min; 3 cm) are depicted by symbols that are colored either red (open and filled), pink, or purple. The data obtained in the absence of LED illumination are shown by symbols that are colored either cyan, dark blue (open and filled), or green. Cellular [^33^P]-Pi uptake was determined at 10 min times points as described previously.^5^ Data from 3 biological replicates are presented as means ± standard deviations.

In a recent study^5^ we pursued PP-InsP functionality using WT HCT116 cells and a subline that is 1,5-InsP_8_-null (*i.e.*, PPIP5K–KO; Fig. 1); in that work, we demonstrated that these KO cells exhibited an approximately 20% decrease in the rate of Pi uptake compared to WT cells. Similar data were obtained in the current study (Fig. 8A–C). This impact on Pi uptake was previously shown to be phenocopied by KO of the Xenotropic and Polytropic Retrovirus Receptor 1 (XPR1);^5^ we have hypothesized that 1,5-InsP_8_ enhances Pi uptake through direct, allosteric interactions with XPR1. However, in that study we did not study rapid cellular actions of 1,5-InsP_8_; instead, we delivered PP-InsPs into cells inside an alternative liposome formulation that degraded spontaneously over a 3 h time period. Moreover, to avoid complications that might arise from 1,5-InsP_8_ metabolism, we previously used metabolically stable 1,5-PCP-InsP_8_; its delivery into PPIP5K–KO cells rescued the defect in Pi uptake.^5^

We have now used our new delivery method to answer different questions: what is the impact of the natural 1,5-InsP_8_ signal, can it act rapidly (*i.e.*, within minutes), and does it regulate Pi uptake into WT cells? Thus, various concentrations of liposomes that contained 1,5-InsP_8_ were added to wild-type WT HCT116 cells incubated at 37 °C for 4 h. Then, we irradiated the cells for 5 min to release the encapsulated cargo (Fig. 8A), and immediately performed a [^33^P]-Pi uptake assay, which was quenched after 10 min (Fig. 8B). We also performed control experiments: there was no effect upon Pi influx in cells loaded with empty liposomes, either with LED illumination (pink symbols in Fig. 8B–E) or without illumination (cyan symbols in Fig. 8B–E). There was also no effect on Pi influx in cells loaded with 1,5-InsP_8_ in the absence of LED illumination (dark blue symbols in Fig. 8B and C). However, we found that the liposomes loaded with 1,5-InsP_8_ that was released by the LED stimulated the uptake of [^33^P]-Pi in a manner that is dependent upon the quantity of liposomes that were added; cell treatment with 40 μg mL^–1^ liposomes doubled the rate of Pi uptake (Fig. 8B). These new data offer a new perspective on the regulation of cellular Pi uptake by 1,5-InsP_8_ compared to our previously-published results,^5^ which focused on the decrease in the rate of Pi uptake into PPIP5K KO cells. That is, our new experiments illuminate a wider rheostatic range of concentrations over which 1,5-InsP_8_ is functional, by showing for the first time a dose-dependent stimulation of Pi uptake as 1,5-InsP_8_ levels are elevated beyond steady-state levels in WT cells (which occurs, for example, following stimulation of L6 myoblasts with PDGF, or insulin^53^). Thus, our data enhance our understanding of the quantitative importance of 1,5-InsP_8_ to Pi homeostasis. Consequently, it now becomes plausible to consider that perturbation of Pi homeostasis (which unbalances countless metabolic processes^54^), offers a mechanistic basis by which excess 1,5-InsP_8_ production is associated with the deafness and keratoconus phenotypes that have been linked to certain PPIP5K missense mutations.^55^,^56^

It is also notable that the addition to PPIP5K KO cells of 30–40 μl mL^–1^ of liposomes that contained 1,5-InsP_8_ restored the degree of Pi uptake to the level observed for untreated WT cells (Fig. 8B and C). In control experiments, liposomal delivery of InsP_6_ was ineffective (Fig. 8F and G). We also found the delivery of 1,5-PCP-InsP_8_ into WT and PPIP5K KO HCT116 cells stimulated Pi uptake in a manner that was quantitatively similar to that elicited by 1,5-InsP_8_ (Fig. 8B–E). This is the first quantitative comparison of the actions of 1,5-InsP_8_ and 1,5-PCP-InsP_8_ in a cell-based bioassay, and as such our data testify to the value of this analogue as a functional bioisostere when studying rapid signaling events that do not involve protein pyrophosphorylation.^2^

### Concluding comments

The LED-initiated, thermolytic release of liposome-delivered PP-InsP into intact cells adds a significant new tool for studying cell signaling by this family of signaling molecules. In the absence of specific inhibitors of PPIP5K-mediated 1,5-InsP_8_ synthesis, most previous advances into the biological activities of this particular PP-InsP have primarily been driven by long-term genetic perturbations of PPIP5K expression.^11^,^31^,^57^–^60^ In contrast, we have described a method that can illuminate molecular actions of 1,5-InsP_8_ within a time-frame of a few minutes. Our methodology uses near infra-red irradiation which is more compatible with biological samples than is UV irradiation in uncaging experiments, which can generate damaging radicals. It is also significant that the use of a 22 mm collimated beam from an LED has key advantages over a laser: the LED can illuminate a larger number of cells (10^6^ cells in a 12-well dish), it has no safety issues, and it facilitates consistency of application between different laboratories. We have also shown the flexibility of this methodology to deliver multiple alternate polyphosphates: 1,5-InsP_8_, 1,5-PCP-InsP_8_ and InsP_6_. Finally, we believe the description of the application of click chemistry to the synthesis of 5-FAM-InsP_7_ offers a new and versatile PP-InsP analogue, and the novel synthetic pathways illuminate a gateway to the preparation of other novel molecules to facilitate further PP-InsP research.

## Materials and methods

### Materials

All lipids were purchased form Avanti Polar Lipids, Inc. (Alabaster, AL). Chloroform was obtained from Fisher Scientific. The 1,1′-dioctadecyl-3,3,3′,3′ tetramethylindotricarbocyanine iodide (DiR) was purchased from AAT Bioquest Inc. HEPES was purchased from Affymetrix, Inc. TiO_2_ beads were obtained from GL Sciences Inc. The 1,5-InsP_8_ and 1,5-PCP-InsP_8_ were synthesized as previously described.^34^,^46^ The synthesis of 5-FAM-InsP_7_ began with a fluorenylmethyl derivative of InsP_6_ in which the only non-protected phosphate is at the 5-position.^22^,^48^ The latter was phosphitylated with phosphoamidate and oxidized with *meta*-chloroperoxybenzoic acid following a strategy that proceeds *via* P(iii)–(v) intermediates.^46^,^47^ Both reactions proceeded with a yield of 50% underlining the efficiency of the approach. Next, the alkyne modified product was used in an azide–alkyne Huisgen cycloaddition under copper catalysis resulting in the fluorescein modified FAM-5-InsP_7_ product with a yield of 93% (see ESI†). The final preparation of 5-FAM-InsP_7_ was validated by PAGE^62^ and by NMR (see ESI†). The origin of the WT and PPIP5K KO HCT116 cells has been described previously.^63^ The culture medium for these cells was DMEM/F12 plus 10% FBS (Germini Bio-Product) and 100 U mL^–1^ penicillin–streptomycin (ThermoFisher Scientific) at 37 °C with 5% CO_2_.

### Preparation of thermosensitive liposomes

The thermosensitive liposomes were synthesized by a modification of previous procedures.^24^ A mixture of DPPC, DOPC and DiR (9 : 1 : 1 by weight) was dissolved in chloroform and dried under vacuum for 2 h. The resulting lipid film was hydrated with 2 mL of 5 mM of HEPES buffer containing either 100 μM 5-FAM-InsP_7_, or 50 μM 1,5-InsP_8_, or 50 μM mM 1,5-PCP-InsP_8_, followed by five freeze/thaw cycles. The liposome suspension was then extruded through the membrane filters of pore sizes 0.45 and 0.20 μm for 20 times, respectively, and stored for up to one month at 0–4 °C. The encapsulation efficiency was approximately 20%; prior to each experiment, liposomes were centrifuged (17 000 × *g*; 1 h; 0–4 °C) to separate them from non-encapsulated cargo. The photothermal properties of these vesicles *in vitro* were monitored with a thermocouple (product 1235C59, Thomas Scientific). In addition, thermal images were recorded with a FLIR E8 camera (FLIR Systems, Inc.). The hydrodynamic size, PDI and ζ-potential of the liposomes (40 μg mL^–1^ in PBS or DMEM (+10% FBS), as indicated) were all recorded by using a Zetasizer Ultra (Malvern Panalytical).

### Transmission electron microscopy (TEM)

Liposome suspensions were dropped onto a TEM copper grid (300 mesh) (Ted Pella) and then stained with phosphotungstic acid (1%, v/v). After air-drying, the sample was imaged using a Gatan Orius SC 1000 side mount camera attached to a FEI Company Techani T12 TEM operated at 80 kV.

### PAGE analysis of delivery of liposome into cells

Two 150 mm plates, each containing approximately 5 × 10^7^ cells cultured in 8 mL medium, were incubated with 120 μg mL^–1^ liposomes for 6 h. Following acid-quench^63^ we concentrated polyphosphates with TiO_2_ beads, and analyzed their levels by PAGE.^64^ For this analysis, 31.7% polyacrylamide gels were freshly prepared from the following: 55.5 mL of 19 : 1 (v/v) 40% acrylamide : bis-acrylamide (IBI Scientific), 7 mL of 10× Tris/Borate/EDTA buffer, 7.5 mL H_2_O, 262 μl ammonium persulfate, and 30 μl tetramethylethylenediamine. The running buffer was 0.5× Tris/Borate/EDTA. A Hoefer tall standard dual cooled vertical unit was used as the electrophoresis system. The gel was pre-run for 1 h at 200 V, and then the gel lanes were loaded with cell extracts (50 μl; prepared as described above) plus 12.5 μl of 6× orange G dye. The gel was run at 300 V for 1 h until the dye front entered the gel. The gel was then run at 500 V for 20 h. Gels were stained for 30 min with a mixture of 0.05% (w/v) toluidine blue (Sigma Aldrich), 20% (w/v) methanol, 2% (w/v) glycerol in water, and then de-stained for 2 h with 2 and 3 changes of 20% (w/v) methanol, 2% (w/v) glycerol in water.

### Flow cytometry

A BD LSR II flow cytometer was used to assay 5-FAM-InsP_7_ fluorescence (excitation = 488 nm; emission = 530/30 nm) at 25 °C in single live cells gated by standard procedures. Unsorted fluorescence intensity data were plotted using FlowJo (v10).

### Confocal microscopy

Live cells were stained with 50 nM LysoTracker Deep Red (Invitrogen) at 37 °C for 20 min. The cells were then washed with PBS twice and stained with 2 μg mL^–1^ Hoechst 33258 at 37 °C for 20 min. After further washing with PBS, the cells were immediately analyzed by confocal microscopy. Images were taken with a Zeiss LSM780 (Carl Zeiss Inc, Oberkochen, Germany) using the following excitation/emission pairs: 405 nm/415–491 nm (for Hoechst); 488 nm/491–571 nm (for 5-FAM-InsP_7_) and 594 nm/597–686 nm (for LysoTracker Red). A plan-APOCHROMAT 63×/1.4 Oil DIC objective was used, along with a pinhole setting which yields an optical slice of 0.9 μm. The cell numbers were counted using the SPOT function in Imaris software (v 9.2.0).

Weighted colocalization coefficients were calculated using the Zeiss Zen Black software, to describe the number of green pixels (5-FAM-InsP_7_) that colocalize with red pixels (LysoTracker), divided by the total number of red pixels.

### Cell cytotoxicity assay

An MTT assay was used to determine the cytotoxicity of liposomes upon LED irradiation. HCT116 cells were randomly seeded into 96-well plates at a density of 5000 cells per well (100 μL), under 100% humidity and were cultured at 37 °C with 5% CO_2_ for 48 h. The indicated concentration of liposomes was added to the cells for 4 h, after which the cells were replenished with fresh medium and irradiated with the LED. After further incubation for 24 h, 10 μL of MTT solution (5 mg mL^–1^) was added to each well to a final volume of 100 μL. After that, the plate was placed in the CO_2_ incubator for additional 4 h. The media was removed and DMSO (100 μL) was added into each well. Absorbance values were determined with synergy 2 multi-detection microplate reader (BioTek) at 570 nm. The cell viability was estimated according to the following equation: cell viability (%) = (OD_treated_/OD_control_) × 100.

### LED irradiation

For our studies we used an “ultra high-power” LED (model UHP-T-730-LA; Prizmatix, Givat-Shmuel, Israel) with tunable power output from 0 to 900 mW, a wavelength peak at 734 nm (±20 nm at 50% power output), and a collimated beam diameter of 22 mm. The distance from the lamp to the sample was adjusted as described in the figures.

### Phosphate uptake assay

WT and PPIP5K KO cells were seeded in 12-well plates. Pi transport assays were performed 48 h later at 37 °C according to a previously published protocol^65^*i.e.*, the culture medium was replaced with phosphate-free DMEM (Gibco catalog number: 11971-025) and 0.5 μCi mL^–1^ of either [^33^P]-Pi (ARP 0153, ARC) was added. At the indicated times, cells were separated from medium by three rapid washes in 1 mL of ice-cold Pi-buffered saline (PBS). Cells were then lysed in 0.5 mL PBS plus 1% Triton X-100, and the accumulated [^33^P]-Pi was determined using a liquid scintillation counter. Uptake was normalized to cell protein, as determined using a BCA protein assay (Pierce).

## Contributions

Z. W., H. W., H. J. J. and S. B. S. led project design. Z. W., N. J. and T. B. performed experiments. Z. W., H. J. J. and S. B. S. wrote the manuscript. All authors have approved the submission.

## Conflicts of interest

The authors declare there is no conflict of interest.

## Supplementary Material

Supplementary information
