# Supplementary material for: Rapid stimulation of cellular Pi uptake by the inositol pyrophosphate InsP8 induced by its photothermal release from lipid nanocarriers using a near infra-red light-emitting diode
Source: Chem Sci. 2020 Sep 8;11(37):10265–78. doi: 10.1039/d0sc02144j (PMC7891704; doi:10.1039/d0sc02144j)
Supplement: Supplementary file 1 [file SC-011-D0SC02144J-s001.pdf]

Electronic Supplementary Material (ESI) for Chemical Science.
This journal is © The Royal Society of Chemistry 2020

Supplementary Information: Chemical syntheses

Reactions were carried out using dried glassware under an inert gas atmosphere (Ar or N_2) and magnetically stirred, unless noted otherwise. Air- and moisture-sensitive liquids and solutions were transferred via a disposable syringe or a stainless steel cannula. Syringes and cannulae were always purged with argon or nitrogen before use.
Reagents were purchased from commercial suppliers and used without further purification, unless noted otherwise.
Solvents were dried with the Braun Solvent Purification System 800 (CH_2Cl_2, Et_2O, THF) by the technical service of the University of Freiburg, or used from commercial suppliers. All dry solvents were stored over molecular sieves (3 or 4 Å) and under inert atmosphere.
Thin layer chromatography (TLC) was performed on Merck silica gel 60 F254 plates (0.25 mm layer thickness, with fluorescence indicator). TLC was visually analyzed by UV (λ = 254 nm) and stained with $KMnO_4$ solution (2.00 g $KMnO_4$, 4.00 g Na_2CO_3, 200 mL dist. H_2O) or phosphomolybdic acid stain (PMA, 3-4 g $H_3PMo_{12}O_{40}$ in EtOH).
Flash column chromatography was carried out using silica gel 60 (0.04 – 0.063 mm, 230 – 400 mesh) of Macherery-Nagel as stationary phase.
NMR spectroscopy: ^1H-, ^{13}C- and ^{31}P-NMR spectra were measured on Bruker Avance III HD 300 MHz, Bruker Avance Neo 400 MHz and Bruker DRX 500 NMR spectrometers in the indicated deuterated solvents. All signals were referred to an internal solvent signal standard (^1H-NMR: $CDCl_3$: δ = 7.29 ppm, D_2O: δ = 4.79 ppm, MeOD-d4: δ = 3.31 ppm, DMSO: δ = 2.50 ppm, ^{13}C-NMR: $CDCl_3$: δ = 77.16 ppm). The signals of ^{31}P-NMR spectra were referenced to an external standard. Data are reported as follow: chemical shift (δ /ppm), multiplicity (s: singlet; d: doublet; t: triplet; hept: septet; m: multiplet), coupling constant(s) (J/Hz), integration. All measurements were carried out at 298 K. The evaluations of the NMR-spectra were made with MestReNova 12.0.1-20560.
High resolution mass spectrometry (HRMS) was performed by the analytical department of the Institute of Organic Chemistry at the University of Freiburg. Measured on Thermo LCQ Advantage (spray voltage: 2.5 – 4.0 kV; spray current: 5 µA; ion transfer tube: 250 (150) °C, evaporation temperature: 50 – 400 °C).

Synthesis of inositol derivative 4

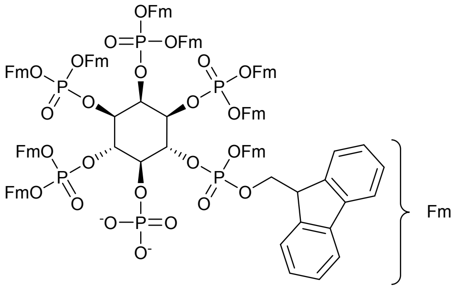

Inositol derivative **4** was synthesized as described before in 6 steps, starting from myo-inositol. Analytical data were identical with the values reported in the literature (1).

Synthesis of P-amidite 5

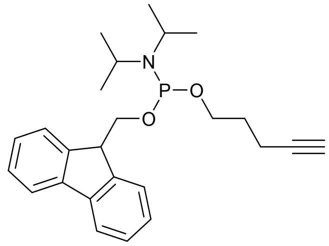

P-amidite (**5**) was synthesized as described before in 2 steps, starting from Bis(diisopropylamino)chlorophosphine. Analytical data were identical with the values reported in the literature (2).

^{1}H-NMR (300 MHz, $CDCl_3$) δ: 7.79 (dd, *J* = 7.6, 1.1 Hz, 2H), 7.70 (dddt, *J* = 12.1, 7.3, 1.5, 0.8 Hz, 2H), 7.47 – 7.38 (m, 2H), 7.33 (tdd, *J* = 7.4, 3.1, 1.3 Hz, 2H), 4.24 (t, *J* = 7.3 Hz, 1H), 3.91 – 3.57 (m, 5H), 2.34 (td, *J* = 7.1, 2.6 Hz, 2H), 1.97 (t, *J* = 2.7 Hz, 1H), 1.86 (tt, *J* = 7.1, 6.1 Hz, 2H), 1.22 (d, *J* = 6.8 Hz, 6H), 1.19 (d, *J* = 6.8 Hz, 6H) ppm; **^{13}C-NMR** (101 MHz $CDCl_3$) δ: 144.97 (d, *J* = 30.4 Hz), 141.42 (d, *J* = 10.1 Hz), 127.47 (d, *J* = 4.0 Hz), 126.92 (d, *J* = 3.9 Hz), 125.45 (d, *J* = 24.6 Hz), 119.87 (d, *J* = 5.8 Hz), 77.30, 68.55, 66.16 (d, *J* = 17.5 Hz), 62.01 (d, *J* = 17.4 Hz), 49.46 – 49.13 (m), 43.11 (d, *J* = 12.4 Hz), 30.34 (d, *J* = 7.2 Hz), 24.76 (d, *J* = 3.3 Hz), 24.69 (d, *J* = 3.1 Hz) ppm; **^{31}P{^{1}H} NMR** (122 MHz, $CDCl_3$) δ: 146.27 ppm; **HRMS:** (ESI) $[M]^+$ calculated for $C_{25}H_{33}O_2NP$: 410.2243, found 410.2243.

Synthesis of inositol derivative 6

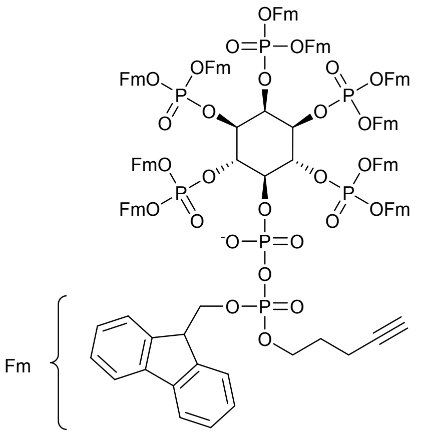

Inositol derivative **4** (188 mg, 77.1 µmol, 1.0 eq.) and P-amidite **5** (63.0 mg, 154 µmol, 2.0 eq) were coevaporated with MeCN (2 mL). The residue was dissolved in CH_2Cl_2 (2.5 mL) and DCI (18.2 mg, 154 mmol, 2.0 eq.) was added. The reaction was stirred for 10 min. and then oxidized by careful addition of *m*CPBA (77% moistened with water, 38.0 mg, 154 mmol, 2.0 eq). The reaction mixture was concentrated *in vacuo* and precipitated with MeOH (10 mL). The precipitate was isolated by centrifugation and washed with MeOH (5 mL). The crude product was dried *in vacuo* and purified by column chromatography (CH_2Cl_2:MeOH 95:5). The target compound was obtained as a white foam (106 mg, 38.3 mg, 50 %) as a mixture of two diastereomers. **R_f** (CH_2Cl_2: MeOH 95:5): 0.27; **^{31}P{^{1}H} NMR** (122 MHz, Chloroform-*d*, integrals are reported as the sum of two diastereomers) δ: -1.44 (s, 2 P) , -2.04 (s, 1 P), -2.49 (s, 2 P), -10.39 (s, 1 P), -12.19 (s, 1 P) ppm; **HRMS** (ESI) [M]+ calculated for $C_{164}{}^{31}CH_{134}O_{27}P_7$: 2764.7315, found 2764.7244.

Synthesis of inositol derivative 7

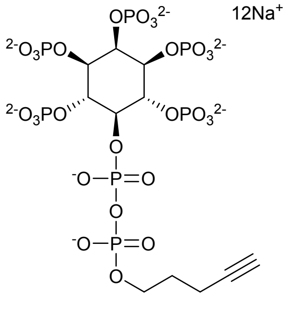

Inositol derivative **6** (53.0 mg, 19.2 µmol, 1.0 eq.) was dissolved in DMF (1.7 mL) and piperidine (300 µL) was added. The reaction mixture was stirred 1 h at r.t. The product was precipitated with Et_2O (18 mL) and separated by centrifugation. The precipitate was dissolved in MeOH (500 µL) and crystallized by addition of cooled $NaClO_4$ solution (0.5 M in acetone, 1 mL). Centrifugation process was repeated and yellowish crystals were obtained which were washed with cold acetone (500 µL). The solid was dissolved in H_2O (100 µL), precipitated with MeOH (1.4 mL) and separated by centrifugation. The target compound was obtained as colourless crystals (10.2 mg, 9.54 µmol, 50%). **^{1}H NMR** (400 MHz, D_2O, the signal of

one Inositol proton is overlain by the solvent peak) δ: 4.50 – 4.39 (m, 1H), 4.30 – 4.19 (m, 1H), 4.14 – 4.05 (m, 2H), 4.02 (q, *J* = 6.4 Hz, 2H), 2.31 – 2.24 (m, 3H), 1.81 (p, *J* = 6.7 Hz, 2H). ppm; **^{31}P{^{1}H} NMR** (162 MHz, D_2O) δ: 1.87 (s, 2 P), 1.14 (s, 1 P), 0.45 (s, 2 P), -10.15 (d, *J* = 16.9 Hz), -10.64 (d, *J* = 16.7 Hz) ppm; **HRMS** (ESI) $[M]^{3-}$ calculated for $C_{11}H_{22}O_{27}P_7$: 267.6176, found 267.6176.

Synthesis of inositol derivative 9

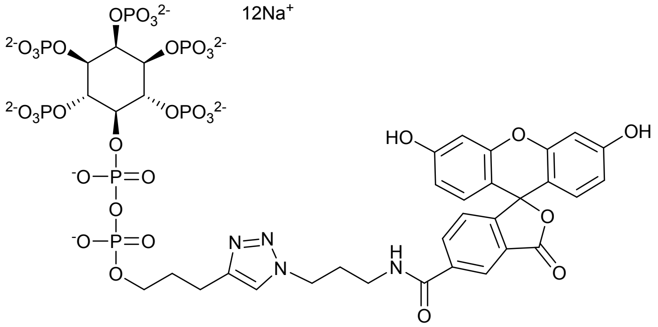

Inositol derivative **7** (5.00 mg, 4.68 µmol, 1.0 eq.) was dissolved in triethylammonium acetate buffer (0.1 M, pH 7, 2 mL). The mixture was degassed by bubbling a stream of argon through the solution for 10 min. Subsequently, a solution of 5-FAM-azide (**8**, 3.23 mg, 7.02 µmol, 1.5 eq.) in DMSO (100 µL), $CuSO_4 \times 5\ H_2O$ (1.17 mg, 4.68 µmol, 1.0 eq.) and sodium ascorbate (4.63 mg, 23.4 µmol, 5.0 eq.) were added and the reaction mixture was stirred under argon atmosphere for 3 h. Afterwards, a solution of $NaClO_4$ (0.5 M in acetone 2 mL) followed by acetone (5 mL) were slowly added at 0 °C to form a precipitate. The precipitate was isolated by centrifugation, washed with acetone (1 mL) and dried under high vacuum. The product was obtained as orange-red crystals (6.65 mg, 4.35 µmol, 93%). **^{1}H-NMR** (400 MHz, D_2O) δ: 8.21 (d, *J* = 1.9 Hz, 1H), 7.98 (dd, *J* = 8.0, 1.9 Hz, 2H), 7.95 (s, 1H), 7.50 (d, *J* = 7.9 Hz, 1H), 7.22 (dt, *J* = 9.6, 1.2 Hz, 2H), 6.69 (d, *J* = 1.1 Hz, 3H), 6.67 (d, *J* = 2.3 Hz, 1H), 4.58 (t, *J* = 6.9 Hz, 2H), 3.76 – 3.69 (m, 1H), 3.68 – 3.61 (m, 1H), 3.52 (t, *J* = 6.5 Hz, 4H), 2.86 (t, *J* = 7.7 Hz, 2H), 2.25 – 2.23 (m, 3H) ppm; **^{31}P{^{1}H} NMR** (202 MHz, D_2O) δ: 3.76 – 0.18 (m, 5P), -9.03 – -12.02 (m, 2P) ppm; **HRMS** (ESI) $[M]^{3-}$ calculated for $C_{35}H_{40}O_{33}N_4P_7$: 420.3252, found 420.3252.

^{31}**P-NMR** (122 Hz) of **6** in $CDCl_3$.

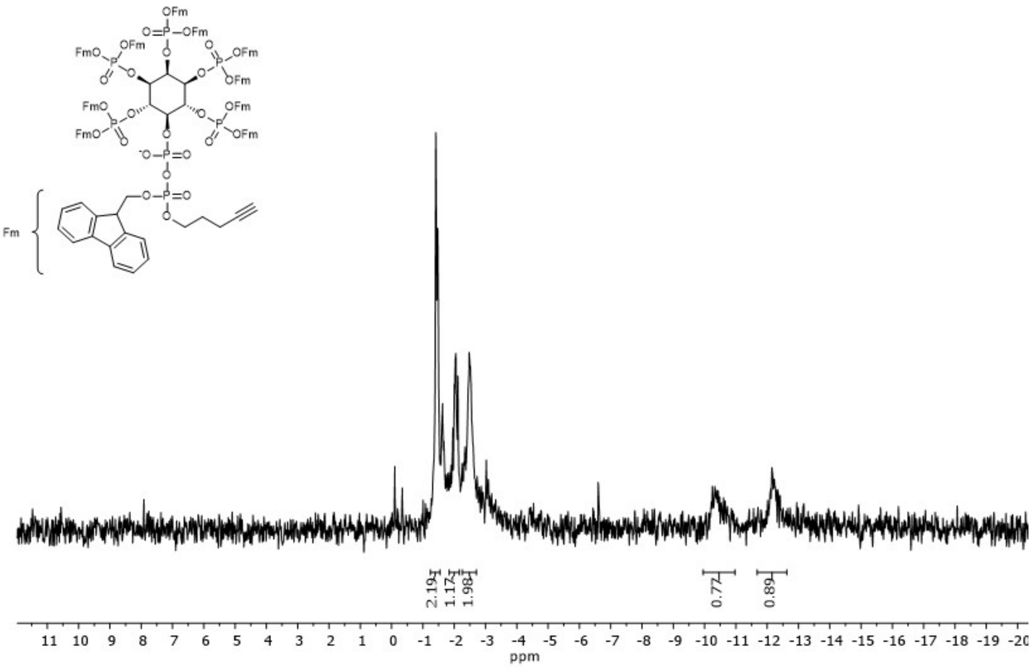

HRMS (ESI+) of **6**

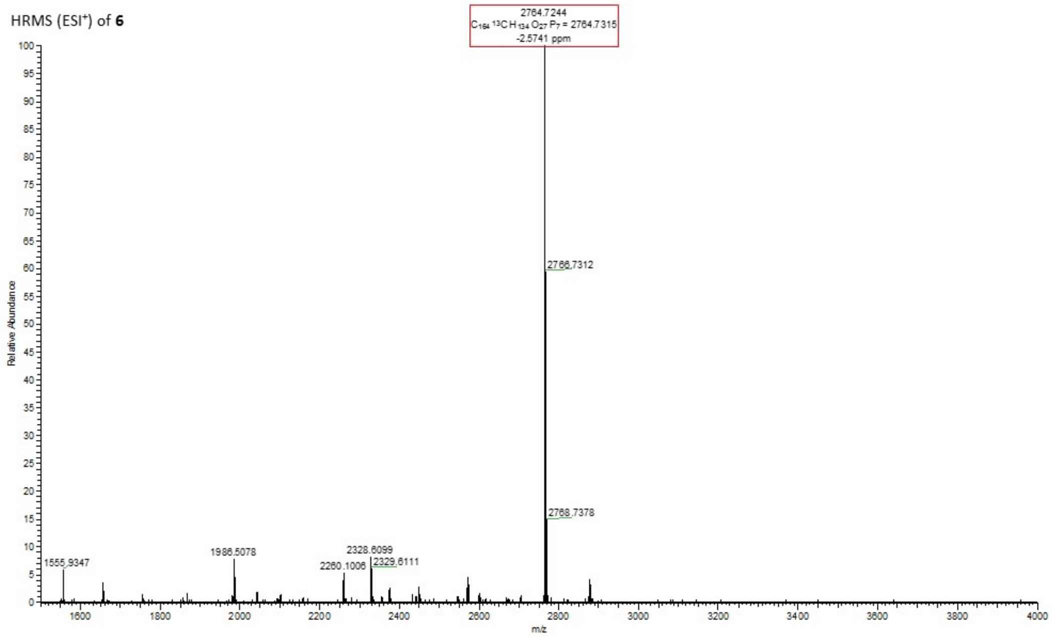

¹H-NMR (400 Hz) of **6** (as a mixture of diastereomers) in $CDCl_3$.

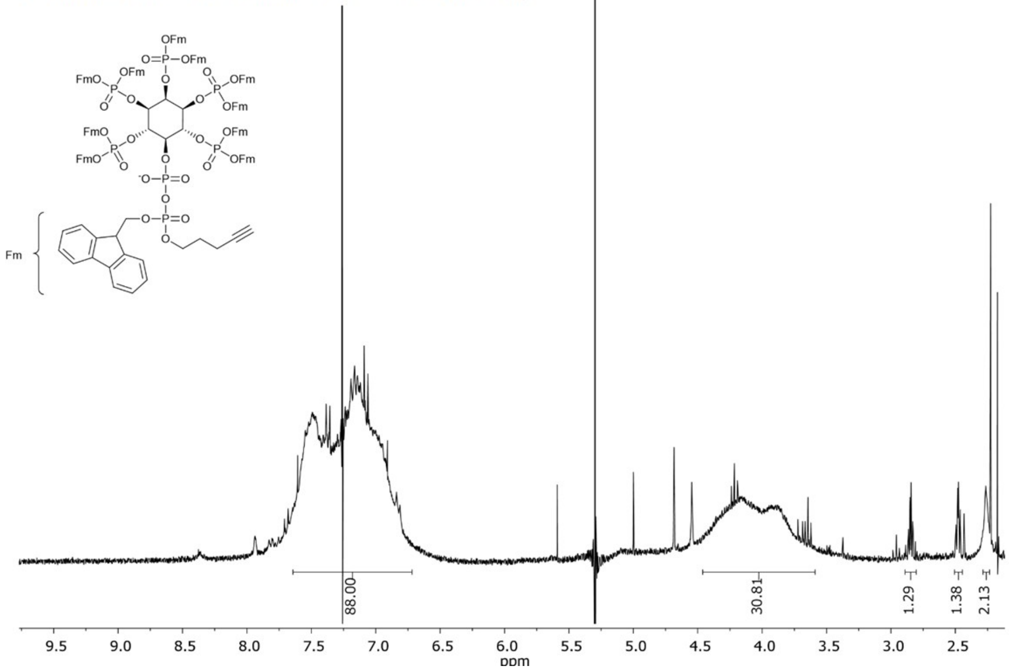

¹H-NMR (400 Hz) of **7** in D_2O.

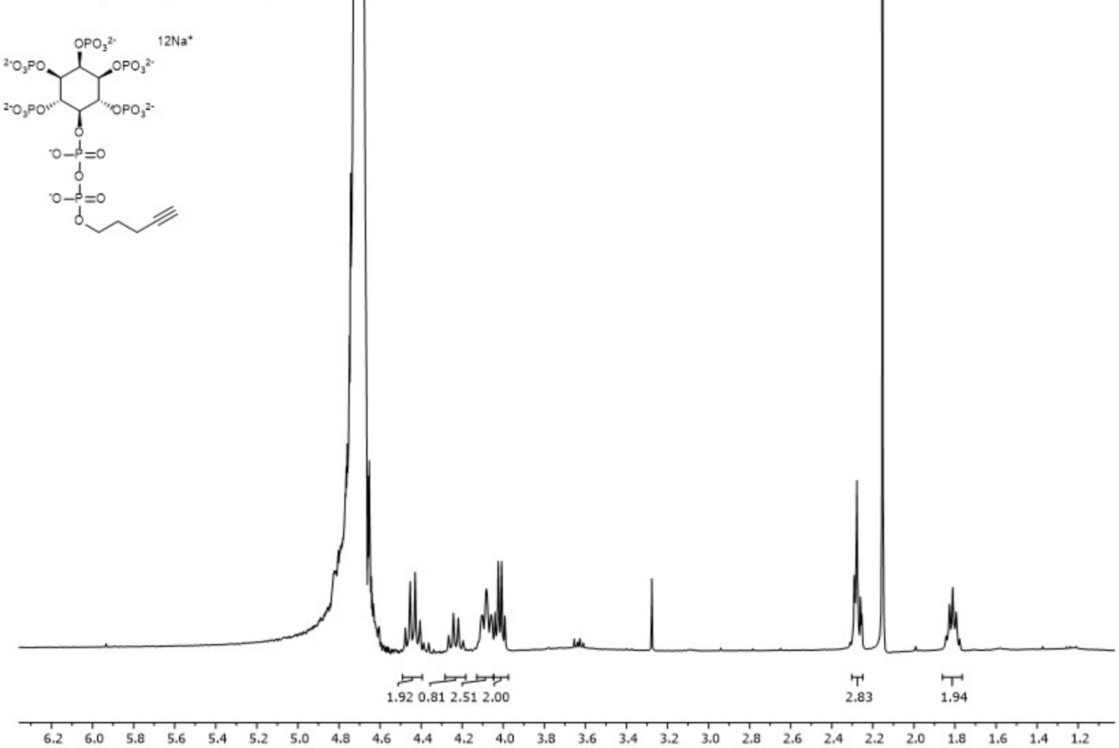

^{31}**P-NMR** (122 Hz) of **7** in D_2O.

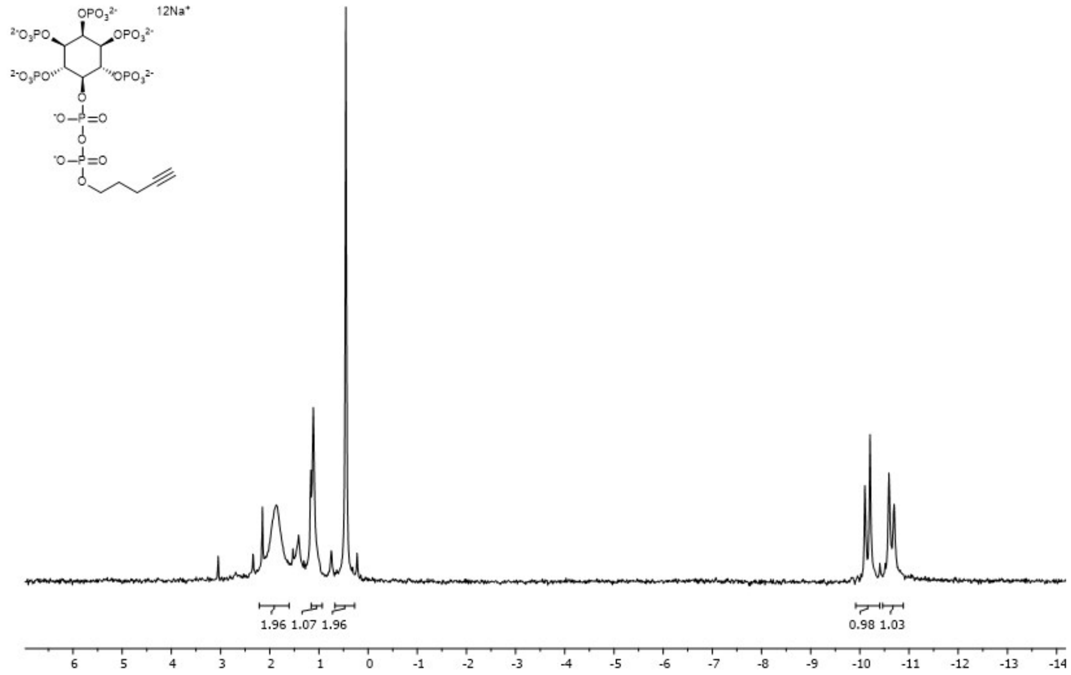

HRMS (ESI$^+$) of **7**

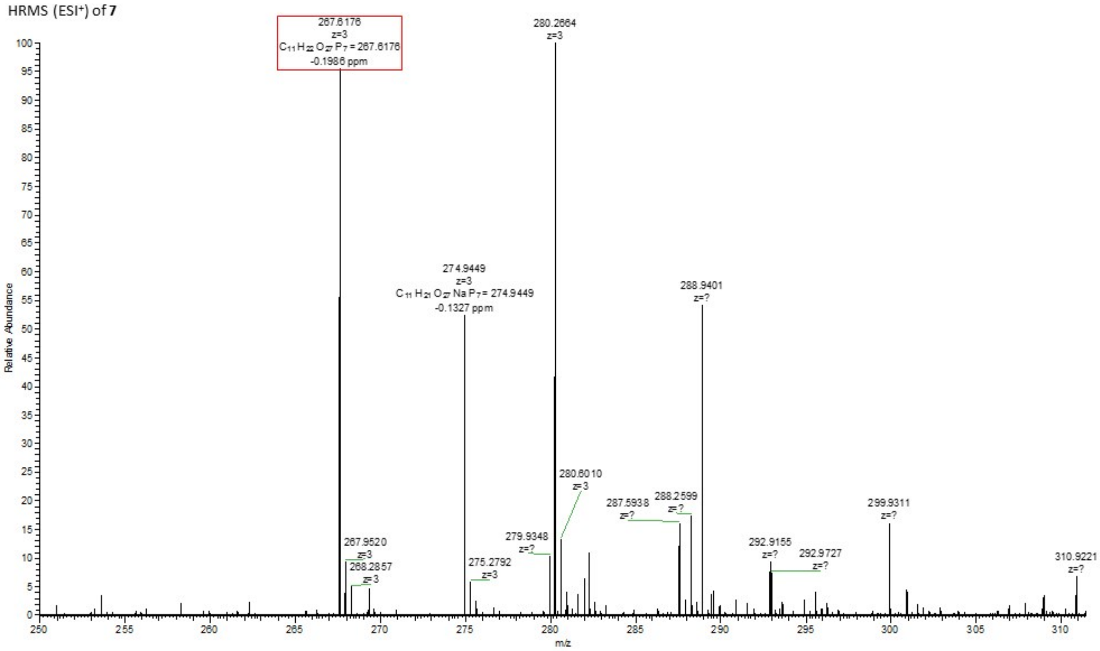

^{1}**H-NMR** (400 Hz) of **9** in D_2O.

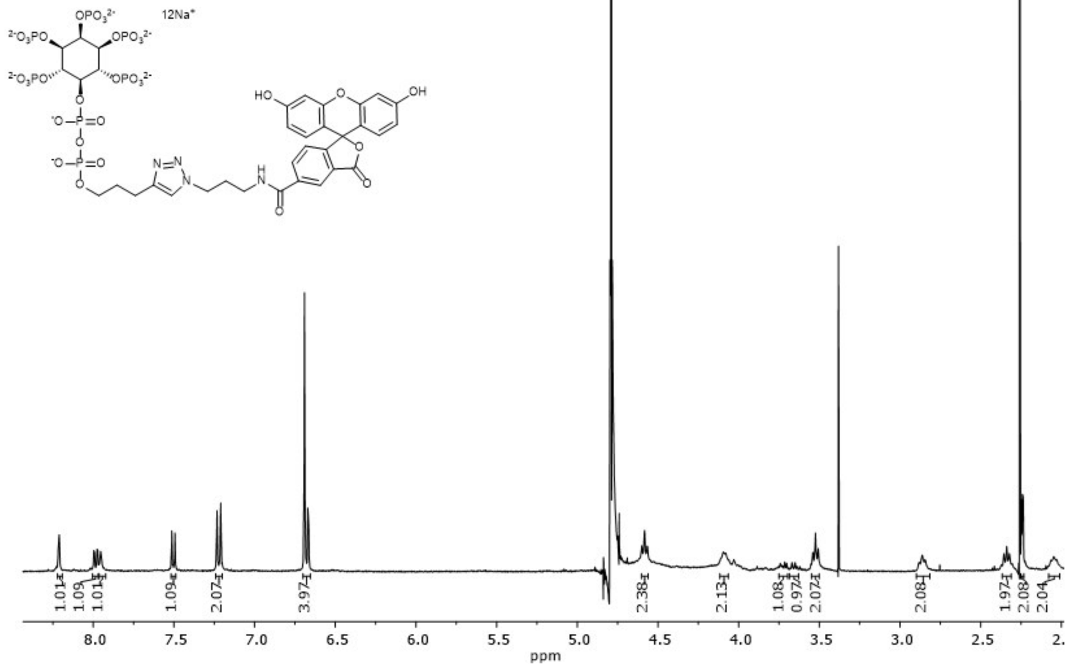

^{31}P-NMR (122 Hz) of **9** in D_2O.

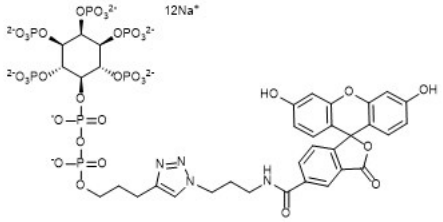

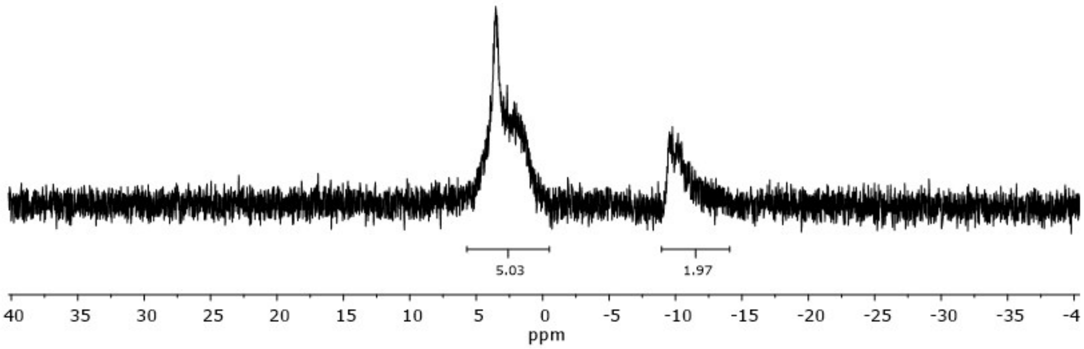

HRMS (ESI+) of **9**

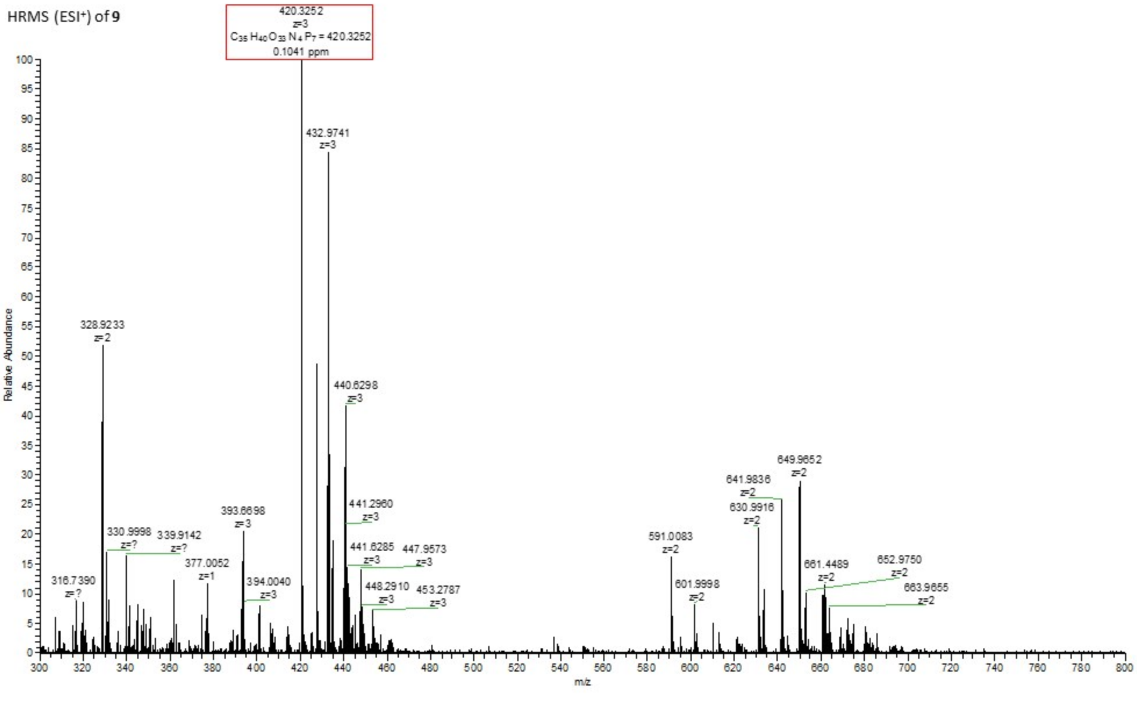

1. I. Pavlovic *et al.*, Cellular delivery and photochemical release of a caged inositol-pyrophosphate induces PH-domain translocation in cellulo. *Nat. Commun* **7**, 10622 (2016).
2. T. M. Haas *et al.*, Magic spot nucleotides: tunable target-specific chemoenzymatic synthesis. *Chem Commun (Camb)* **55**, 5339-5342 (2019).
